# Antipsychotic Medication Preferences: A Large-Scale Survey

**DOI:** 10.1055/a-2784-3444

**Published:** 2026-02-19

**Authors:** Babet N. Wezenberg, Jara Bouws, Marieke van der Pluijm, Mariken B. de Koning, Martijn Kikkert, Matthijs Blankers, Iris E. Sommer, Floor A. van Dijk, Lieuwe de Haan

**Affiliations:** 1 26066 Department of Psychiatry, Amsterdam UMC Location University of Amsterdam, Amsterdam, The Netherlands; 2 Department of Research, Arkin Mental Health Care, Amsterdam, The Netherlands; 3 3647 Department of Biomedical Sciences of Cells & Systems, Cognitive Neurosciences, University of Groningen, University Medical Center Groningen (UMCG), Groningen, The Netherlands; 4 Department of Research, Delfland Mental Health Care, Delft, The Netherlands

**Keywords:** Psychosis, antipsychotic drugs, survey, preferences

## Abstract

**Introduction:**

Antipsychotic medications are essential in treating psychotic disorders; yet, large-scale studies examining individual patient preferences are lacking. The current study explores variations in patient perspectives on antipsychotic treatment.

**Methods:**

Data were derived from the online decision aid Personal Antipsychotic Choice Index in the Netherlands. This freely available 30-item questionnaire assessed preferred treatment effectiveness, side effects, administration routes, and demographics, psychosis history, and antipsychotic use. Non-parametric tests compared responses by sex, age, and D _2_ -receptor affinity of used medication.

**Results:**

Of 23,733 completed questionnaires, 8,743 individuals with at least one psychotic episode were included (mean age: 38.8 and male: 58.8%). Psychotic symptom efficacy was rated most important (75.4%) followed by cognitive (61.9%) and depressive (49.5%) symptom efficacy (both *p* <0.001). Women rated psychotic and depressive symptom efficacy more important than men ( *p* =0.003 and *p* <0.001), while adolescents prioritized cognitive symptom efficacy over adults ( *p* =0.004). Extrapyramidal symptoms (73.2%) and weight gain (68.0%) were least acceptable side effects, particularly among women (both p<0.001). Men found sexual dysfunction less tolerable than women ( *p* <0.001). Daily tablets were considered most acceptable (91.0%), followed by weekly tablets (40.1%), injectables (32.7%), and drops (27.9%). Patients using low D _2_ -affinity agents were more willing to accept dizziness ( *p* =0.002) and hypersalivation ( *p* <0.001) than high-affinity users, who in turn accepted sexual dysfunction and menstrual problems more often (both *p* <0.001).

**Discussion:**

Patient preferences regarding antipsychotic medication vary widely. Incorporating these perspectives through shared decision-making may improve trust and adherence.

## Introduction

Psychosis represents a significant global health concern, with an annual incidence rate between 15 and 30 cases per 100,000 individuals. 1​ Individuals experiencing psychosis are typically treated with antipsychotic medications. Medication discontinuation and non-adherence are prevalent in any form of treatment, with an overall incidence rate of 50% according to the World Health Organization. 2​ In the context of major psychiatric disorder patients, 49% of patients are non-adherent. 3​ Among individuals with psychosis, even more than half of the first episode psychosis patients show a period of non-adherence or refuse pharmacological treatment. 4​ Given that discontinuing antipsychotic medication is associated with an increased risk of relapse and increased symptom severity, improving treatment adherence is a key priority in the long-term management of psychotic disorders. 5​ 6​ ​7​ Medication adherence is attributable to multiple factors, including socio-demographic characteristics, symptom severity, illness insight, and side-effects, but perhaps even more important is perceived effectiveness and overall treatment experience. 4​ 8​​ 9​ 10​ ​11​

Shared decision making has been shown to enhance treatment satisfaction, adherence, and quality of life. 12​ 13​ 14​ ​15​ The majority of patients with psychotic disorders retains the ability to make informed and rational decisions regarding important treatment outcomes. 16​ ​17​ Even when impairments in decisional capacity are present, during periods of severe symptoms, they can be diminished through interventions that simplify information and encourage shared decision making. Providing accessible, understandable, and high-quality information about antipsychotic medications is therefore not only a way to enhance overall treatment quality, but also a fundamental right for individuals with psychotic vulnerability. As such a resource did not yet exist in the Dutch language, our research group developed a Dutch decision aid for the selection of antipsychotic medications, designed to involve patients in their treatment decisions and facilitate open discussions with their healthcare professional: the Personal Antipsychotic Choice Index (PACindex or PAKwijzer, www.pakwijzer.nl ). 18​ In the PACindex, patients specify their preferences regarding desired effects, common side effects, and ways of administration. Based on these patient-reported preferences, the PACindex generates a personalized recommendation for antipsychotic medications that best aligns with these preferences. The data collected through the PACindex provides a unique opportunity to gather empirical evidence on the specific preferences of patients, including subgroups within this population, regarding antipsychotic treatment.

The primary objective of the current manuscript is to evaluate which effect and side effect respondents consider important when using antipsychotic medications. Using data from the PACindex, this represents the first large-scale investigation in the Netherlands to systematically evaluate patient perspectives on the antipsychotic medication use. Previous studies have examined treatment goals and patient perspectives on antipsychotic therapy, showing that both clinicians and patients generally prioritize the reduction of psychotic symptoms, while patients place relatively greater importance on avoiding burdensome side effects such as weight gain. 19​ ​20​ We will examine variations in preferences across sex, age, and dopamine (D _2_ ) receptor affinity of the agents. These factors were selected based on prior evidence by Barbui et al., 21​ Gareri et al., 22​ Kapur et al., 23​ Lieberman. 24​ Research by Barbui, Nose, Bindman, et al. 21​ has shown that sex has a significant impact on the subjective acceptability and tolerability of medications. Similarly, physiological changes associated with age affect drug metabolism and elimination and alter both the efficacy and tolerability of antipsychotic agents. 22​ Additionally, differences in pharmacological profiles of antipsychotic medications have a different impact on both therapeutic benefits and burdens of adverse effects. 23​ ​24​ In light of this evidence, these factors were included to ensure an understanding of how demographic and drug-specific variables interact to shape patient preferences and treatment outcomes. Partly based on these insights, we hypothesized that age or gender influences priority concerning the effect and adverse effects. Specifically, we hypothesize that women and older adults will prioritize to have less adverse effects compared to men and younger adults. Our findings may offer a more comprehensive picture of the diverse preferences of specific patient groups on the effect and adverse effects of antipsychotic medication. This information can add evidence-based, experiential knowledge to clinicians’ expertise and enrich shared decision making on antipsychotic choices. Better alignment between patient preferences and treatment choices may lead to increased satisfaction, improved adherence, and ultimately, reduced symptom severity and fewer relapses.

## Methods

### Setting and data collection

This study involves the secondary use of fully anonymized data collected through the PACindex. The PACindex is developed in collaboration with patients and patient organizations, who identified 20 key items reflecting both positive and negative effects of antipsychotic medications. Subsequently, a systematic literature review was conducted to rank commonly prescribed antipsychotics in the Netherlands on each key item, using an evidence hierarchy and a preference-weighted algorithm. The resulting tool, the PACindex, generates a personalized ranking based on user priorities. A comprehensive description of the development of this digital decision aid is described elsewhere. 18​

The decision aid was accessible online and distributed to healthcare providers, who were encouraged to complete it collaboratively with their patients or to inform patients about its availability. Respondents had been informed that their anonymized data would be stored and information was processed anonymously. As no personally identifiable information was saved, and the analyses caused no risk to respondents, a formal ethical review and informed consent were not required. Additionally, given the clinical and public health relevance of understanding patient preferences for antipsychotic medications on such a large scale, these analyses play an important societal role by contributing to more patient-centered treatment approaches. For the current study, data on the decision aid between June 2015 and July 2024 were analyzed. There were no exclusion criteria, as the decision aid was designed to be accessible to all users. Only data of respondents entering an age between 12 and 90 years and experiencing a psychotic episode were analyzed.

### Assessments

The decision aid comprised 30 questions, employing multiple-choice, Likert scale and open-ended response formats. These questions were intended to gather the following data: (1) demographics (age and sex); (2) history of psychosis; (3) current and past antipsychotic medication use; (4) importance of the effect of the medication on psychotic symptoms, depressive symptoms and cognitive symptoms using a Likert scale: 1=“very unimportant,” 2=“unimportant,” 3=“neutral,” 4=“important,” and 5=“very important”; (5) acceptability of the side effects of the medication (weight gain, sexual dysfunction, drowsiness, increased sleep, extrapyramidal symptoms (EPSs), anticholinergic effects – blurred vision, urinating difficulty, constipation, dry mouth – hypersalivation, nausea, dizziness, tiredness, blunted affect, and menstrual disorder using a Likert scale: 1=“very unacceptable,” 2=“unacceptable,” 3=“neutral,” 4=“acceptable,” and 5=“very acceptable”); (6) preferred mode of medication intake (daily tablets, daily drops, weekly tablets and long-acting injectables (LAIs) using “yes” or “no”); and (7) additional questions (smoking, history of epileptic episodes and pregnancy). For the full decision aid questionnaire, see the Supplementary material (available in the online version only).

### Statistical analyses

All analyses were completed using SPSS version 28.0. 25​ Responses were numerically coded in the database. Descriptive statistics were computed for continuous variables (mean and standard deviation) and categorical variables (frequencies and percentages).

First, respondents’ preferences with regard to the effectiveness of antipsychotic medications and the acceptability of side effects were analyzed and ranked. Next, the acceptability of specific therapeutic effects or side effects was analyzed to determine whether preferences differed, and if they differed by sex or age category. The age was categorized into five groups, ensuring sufficient sample sizes for comparison: adolescents, three adult subgroups, and older adults, (<20, 20–35, 36–50, 51–65, and>65 y; **Supplementary Table S1** , available in the online version only). Finally, differences in preferences were assessed among respondents’ using only one type of antipsychotic medication, categorized as high-affinity D _2_ antagonists, low-affinity D _2_ antagonists, and partial D _2_ agonists ( **Supplementary Tables S2 and S3** , available in the online version only). Non-parametric tests were utilized, including the Mann–Whitney *U* test for comparisons by sex and the Kruskal–Wallis test for comparisons across age categories (since the outcome variable was an ordinal). Missing data were addressed using pairwise exclusion (using cases when analyzing variables with non-missing values), Bonferroni correction was used in each analysis to correct for multiple testing, and statistical significance was defined as *p* <0.05. Corrected *p* -values after Bonferroni correction are displayed in the relevant table.

## Results

### Respondents

In total, the PACindex was accessed 23,733 times. Of these, 6,115 surveys were entirely blank and excluded for analysis. Additionally, 31 respondents did not submit an age between 12 and 90 years. Among the remaining eligible respondents, a total of 8,743 individuals reported having experienced a psychotic episode, either currently or in the past, and data from these individuals were included in current analyses. Table 1 presents the demographic details of the included respondents. The mean age of the included respondents was 38.81 years (standard deviation=14.14). Of this group, 58.8% of respondents were males. A majority (85.2%) had used antipsychotic medications in the past, and 79.5% of respondents were currently using antipsychotic medications at the time of completing the questionnaire. For respondents who reported to have never experienced a psychotic episode ( *n* =5,566), 19.4% reported using antipsychotic medications at the time of survey. We do not have additional information on this last group, and it could be plausible that these are individuals with other indications for psychotropic medication or patients who do not acknowledge their experiences as psychosis.

**Table:** **Table 1** Demographic and clinical characteristics of participants with and without a self-reported history of psychotic episodes

Characteristics	Psychotic episode ( *n* =8,743)	No psychotic episode/unknown Data ( *n* =8,844)	*p* -Value
Demographic information			
Sex, men (%)	5,115 (58.8) ^a^	3,399 (48.1) ^b^	<0.001*
Age (y), mean±SD	38.81±14.14 ^c^	35.97±15.31 ^d^	<0.001*
Antipsychotic medication			
Past antipsychotic use, *n* (%)	7,453 (85.2)	848 (9.6)	<0.001*
Current antipsychotic use, *n* (%)	6,530 (74.7)	2,675 (30.2)	<0.001*
History of high affinity dopamine D _2_ -receptor use, *n* (%)	4,791 (54.8)	489 (5.5)	<0.001*
History of low affinity dopamine D _2_ -receptor use, *n* (%)	5,263 (60.2)	570 (6.4)	<0.001*
History of partial dopamine D _2_ -receptor agonist use, *n* (%)	2,261 (25.9)	228 (2.6)	<0.001*
Current high affinity dopamine D _2_ -receptor use, *n* (%)	2,820 (32.3)	950 (10.7)	<0.001*
Current low affinity dopamine D _2_ -receptor use, *n* (%)	3,225 (36.9)	1,530 (17.3)	<0.001*
Current partial dopamine D _2_ -receptor agonist use, *n* (%)	959 (11.0)	400 (4.5)	<0.001*

Abbreviations: D _2_ , dopamine D _2_ receptor; SD, standard deviation.

^a^ Missing 38, ^b^ Missing 1,776, ^c^ Missing 446, and ^d^ Missing 1,968.

* *p* <0.05, Significant.

### Preferences

**Fig. 1:**
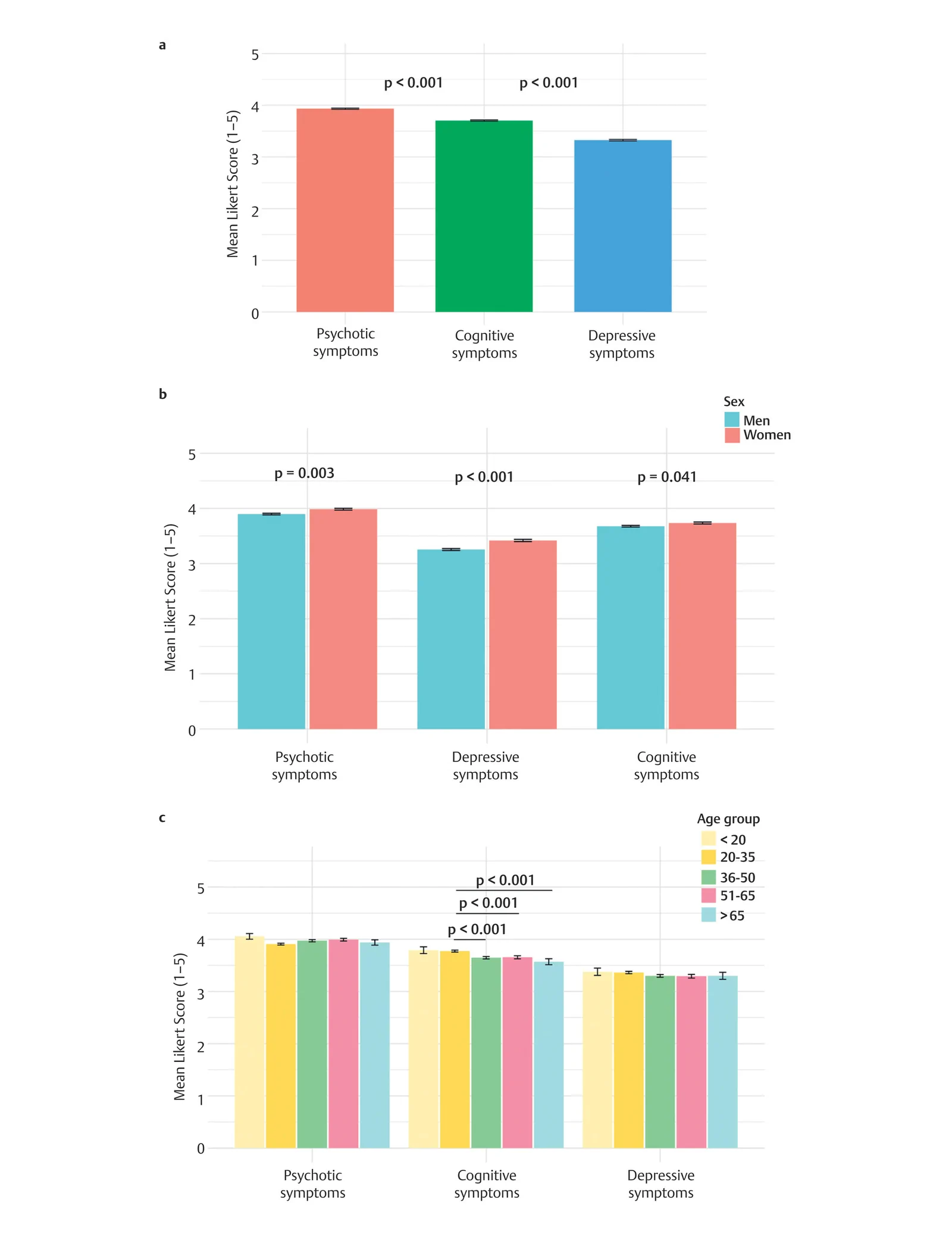
( **a** ) Preferences on effectiveness, in mean Likert-scores and standard errors for total scores. ( **b** ) Preferences on effectiveness, in mean Likert-scores and standard errors for sex differences. ( **c** ) Preferences on effectiveness, in mean Likert-scores and standard errors for age differences. Likert score 1=“very unimportant,” score 2=“unimportant,” score 3=“neutral,” score 4=“important,” and score 5=“very important.”

As shown in Fig. 1a (and **Supplementary Table S4** , available in the online version only), among respondents with psychosis, 75.4% ( *n* =5,920) indicated that it was important or very important because their antipsychotic medication is highly effective in alleviating psychotic symptoms. Additionally, the majority (61.9%; *n* =4,715) emphasized the importance of efficacy for cognitive problems and 49.5% ( *n* =3,692) reported importance for addressing depressive symptoms. These results show marked differences in preferences, with psychotic symptom management being rated significantly higher than cognitive symptom management, and cognitive symptom management being rated significantly higher than the treatment of depressive symptoms ( **Supplementary Table S5** , available in the online version only).

**Fig. 2:**
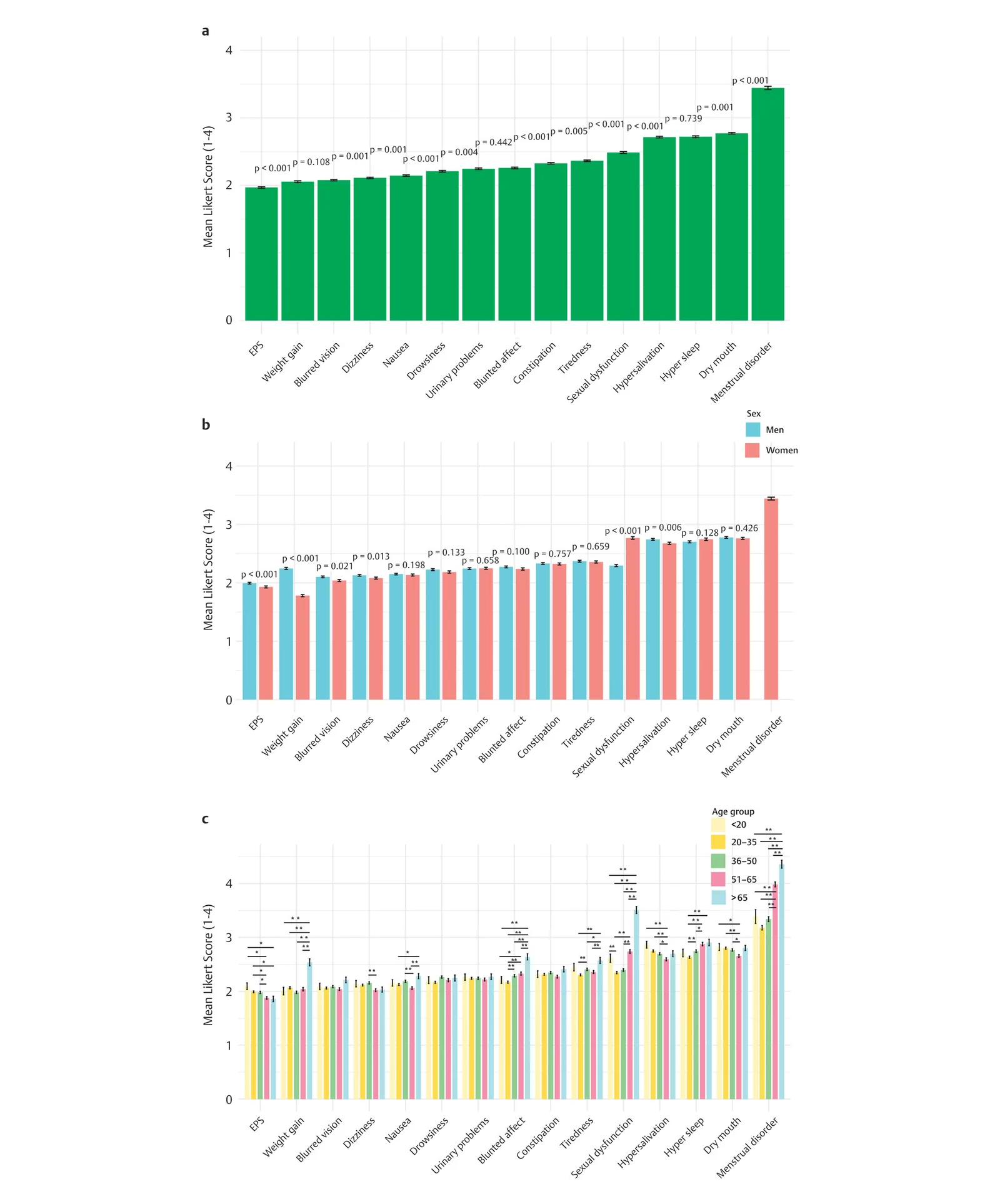
( **a** ) Preferences on side effects, in mean Likert-scores and standard errors for total scores. ( **b** ) Preferences on side effects, in mean Likert-scores and standard errors for sex differences. ( **c** ) Preferences on side effects, in mean Likert-scores and standard errors for age differences. * *p* <0.01, ** *p* <0.001. Likert score 1=“very unacceptable,” score 2=“unacceptable,” score 3=“neutral,” score 4=“acceptable,” and score 5=“very acceptable.”

The order of side effect acceptance displayed a significant difference for nearly every item compared to the following item, indicating that this sequence reflects a representation of the hierarchy of side effect acceptability ( Fig. 2a and **Supplementary Table S6** , available in the online version only). Menstrual disorder and dry mouth were considered the most acceptable side effects. Of all responses on menstrual disorder, filled in only by women ( *n* =2,718), 20.6% ( *n* =561) reported this as (very) unacceptable and then of all responses on dry mouth ( *n* =6,033), 35.2% ( *n* =2,472) reported this as (very) unacceptable. Weight gain and EPSs were considered least acceptable (68.0%, *n* =4,998 and 73.2%, *n* =5,315 respectively, reported this as unacceptable or very unacceptable; **Supplementary Table S7** , available in the online version only).

The preferred mode of medication intake was daily tablets. Ninety-one percentage of respondents reporting experiencing a psychotic episode wanted to take daily medication. The least preferable mode of medication intake was by daily liquid or drops: 27.9% of the respondents was willing to take daily drops ( *p* <0.001; **Supplementary Fig. S1** and **Supplementary Tables S8** and **S9** , available in the online version only).

### Preference differences

#### Sex

Women placed more importance than men on the effectiveness of antipsychotic medication in alleviating psychotic ( *p* =0.003) and depressive ( *p* <0.001) symptoms ( Fig. 1b and **Supplementary Table S10** , available in the online version only).

In terms of side effects, women reported it as more unacceptable to experience EPSs ( *p* <0.001) or gain weight ( *p* <0.001) by their antipsychotic medication than men. Men, on the other hand, reported it more unacceptable to experience sexual side-effects from their antipsychotic medication than women ( *p* <0.001; Fig. 2b and **Supplementary Table S11** , available in the online version only).

#### Age

When comparing age groups regarding the considered importance of effectiveness on symptom categories, only cognitive symptoms differed significantly ( Fig. 1c and **Supplementary Tables S12–S14** , available in the online version only). Adolescents (<20 y of age) reported improvement in cognitive problems more important than 65-year-old or older ( *p* =0.004) and respondents aged 20–35 y reported it more important for their antipsychotic medication to improve their cognitive symptoms compared to all older age categories ( *p* <0.001).

As shown in Fig. 2c , respondents over 65 years of age reported it most acceptable to gain weight, experience nausea, experience a blunted affect, experience more tiredness, and experience sexual dysfunction, increased sleep or menstrual disorders compared to younger age categories ( *p* <0.001). Respondents of younger age categories (<20 y of age and 20–35 y) were more acceptive of EPSs, hypersalivation and dry mouth compared to older age categories ( *p* <0.001–0.013), despite the fact that overall side effects were frequently rated as “very unacceptable.” Respondents in relatively older age categories (36–50, 51–65, and 65>y) accepted the risk of side-effects blunted affect, tiredness, increased sleep and menstrual disorders more often than younger respondents ( *p* <0.001; **Supplementary Tables S15–S17** , available in the online version only).

#### D _2_ affinity

We analyzed respondents with a history or current use of a single type of antipsychotic medication to better capture the origin of specific treatment preferences. Of the 3,798 respondents with a history of using only one type of antipsychotic medication, 42.0% used high affinity D _2_ antagonists, 52.0% used low affinity D _2_ antagonists and 6.0% used partial D _2_ agonists ( **Supplementary Table S2** , available in the online version only for categorization). Of these former users, low D _2_ affinity antagonist users more often preferred antipsychotic medication to improve their cognitive symptoms compared to high D _2_ affinity antagonist users ( *p* =0.016, **Supplementary Fig. S2** and **Supplementary Tables S18–S20** , available in the online version only). Low D _2_ affinity antagonist former users considered it more often acceptable to experience dizziness ( *p* =0.002) or hypersalivation ( *p* <0.001) compared to high D _2_ antagonist users. On the other hand, high D _2_ affinity antagonist former users reported more acceptance to experience sexual dysfunction ( *p* <0.001) and menstrual disorders ( *p* <0.001) compared to low D _2_ affinity users ( **Supplementary Fig. S3** and **Supplementary Tables S21–S23** , available in the online version only).

Of the 6,067 respondents currently using only one type of antipsychotic medication, 40.5% use high affinity D _2_ antagonists, 46.8% use low D _2_ affinity and 12.7% use partial D _2_ agonists. Again, low D _2_ affinity antagonist users preferred antipsychotic medication to improve their cognitive symptoms more often than high D _2_ affinity antagonist users ( *p* =0.002; **Supplementary Fig. S4** , available in the online version only), and partial D _2_ agonist users cared the least about psychotic symptoms improvement compared to high- and low D _2_ affinity antagonist users ( *p* =0.009 and *p* =0.003, respectively; **Supplementary Tables S24–S26** , available in the online version only). Partial D _2_ agonist users reported it to be least acceptable to experience drowsiness ( *p* =0.007 and *p* =0.011) and tiredness ( *p* =0.004 and *p* =0.028) compared to high- and low D _2_ affinity users ( **Supplementary Fig. S5, Supplementary Tables S27–S29** , available in the online version only).

## Discussion

### Main findings

The current study provides insight into the preferences of individuals with a history of, or active psychosis, antipsychotic medications. Several key findings from this study deserve emphasis. First, our results highlight that respondents prioritize the treatment of psychotic symptoms above cognitive symptoms and depressive symptoms, consistent with previous research. 19​ ​20​ Nevertheless, other outcome domains were absent in our questionnaire (i.e., work-related outcomes or personal recovery) which limits our ability to evaluate these patient-centered outcomes. Prioritization of psychotic symptom management is in line with the clinical observation that psychotic symptoms are of most immediate concern and can be directly incapacitating. 26​ ​27​ Lower prioritization of the management of cognitive symptoms could potentially stem from an underestimation of cognitive impairments by individuals with schizophrenia spectrum disorders. 28​ ​29​ While comorbid depression affects 28.6–32.6% of individuals with schizophrenia, 30​ ​31​ the majority does not experience depression, which could explain why respondents rated this aspect as less critical. Another important finding is the general unacceptability of medication side effects. Most side effects were rated as “unacceptable” or “very unacceptable,” which is understandable given the impact of most side effects during long-term treatment. However, the decision aid did not include a question assessing the extent to which individuals would be willing to accept side effects if the medication proved effective in alleviating psychotic or other symptoms. Research indicates that informed consent regarding side effects 32​ contributes to medication adherence in individuals with psychotic disorders. The fact that users of the antipsychotic decision aid demonstrated considerable unacceptability of the mentioned side effects should encourage clinicians to provide thorough, balanced, and transparent information about both the potential benefits and risks of antipsychotic treatment, fostering shared decision-making and improving adherence outcomes.

Consistent with former research, 19​ ​20​ weight gain and EPSs were considered least acceptable; however, blurred vision emerged as the third least acceptable side effect, despite being relatively uncommon clinically. This could display a fear of substantial visual impairment and a lack of familiarity with the actual prevalence and impact of this side effect. Surprisingly, menstrual disorders were identified as the most acceptable side effect in women. Possibly due to the survey wording (“How acceptable is it to you that you would menstruate less?”), women did not understand the potential impact on fertility, which highlights a potential source of response bias and underlines the need for clear communication when assessing preferences, especially given the high prevalence of increased prolactin levels by antipsychotic medication, the underlying mechanism of menstrual irregularities. 33​

Additionally, users of the decision aid showed that daily tablet intake was the preferred mode of medication administration. Previous studies show heterogeneity in the preferred mode of intake, where the daily tablet intake is preferred in some studies, 34​ and in other studies patients favor LAIs. 35​ ​36​ A plausible explanation for this discrepancy could stem from different study populations, for example different levels of prior exposure to LAIs, other stages of the illness (e.g., a lack of familiarity of the burden of daily medication intake by respondents with first-episode psychosis) or experience with relapse, a different history of hospitalization, or other perceived beliefs including the general perception that LAIs are more potent or indicate more severe illness. Another explanation could be the way medication options were presented which could influence responses. Framing by emphasizing autonomy or convenience could lead to different preference patterns. However, the decision aid did not differentiate between first-episode versus recurrent patients and these variables were not explored in the present study; consequently, these are only speculations.

### Preferences according to sex

Women placed greater importance than men on the efficacy of antipsychotic medications in improving psychotic and depressive symptoms. This may reflect sex-based differences in symptomatology, as women with psychotic disorders tend to report higher levels of depressive symptoms, whereas men are more likely to experience negative symptoms. 37​ Pharmacokinetic variations between sexes may contribute to the difference in preferences of side effects, as women generally exhibit greater bioavailability and slower drug elimination rates, leading to elevated plasma concentrations and an increased likelihood of side effects. 38​ 39​ ​40​ Despite these well-documented findings, current treatment guidelines still do not integrate gender-specific factors. We found that women more often reported unacceptability for weight gain and EPSs than men. The variability in preferences for side effects could also reflect gender-based experiences. For example, women are more susceptible to weight gain, diabetes and cardiovascular risk associated with antipsychotic medication use, 39​ ​41​ which could lead to the observed higher unacceptability. Another example, while EPSs like acute dystonia are reportedly more common in men, 42​ other EPSs such as antipsychotic-induced parkinsonism, akathisia, and tardive dyskinesia occur more frequently in women. 42​ ​43​ Men reported sexual side effects to be more unacceptable compared to women. Antipsychotic induced sexual dysfunction is well-documented in both genders; however, increased prolactin levels are present with lower doses in women. 44​ ​45​ However, men report a greater decline in quality of life due to sexual dysfunction 46​ and are more likely to exhibit nonadherence as a result. 47​

### Preferences according to age

Important age-related differences were also identified. Adolescents prioritized the treatment of cognitive symptoms more than other age groups, likely reflecting the critical importance of cognitive functioning during this life phase. Older respondents demonstrated greater tolerance of various side effects, including weight gain, nausea, reduced creativity, tiredness, sexual dysfunction, increased sleep and menstrual disorders. This is in contrast with our initial hypothesis that older individuals would be less accepting to side effects due to their heightened vulnerability. 22​ This increased tolerance could be explained by having more experience with chronic illnesses and long-term medication use, which could potentially foster a more pragmatic perspective on balancing benefits and risks of treatment. Additionally, individuals in a different phase of life may place less emphasis on partnership formation or pursuing educational or occupational roles, possibly resulting in greater tolerance of side effects.

### Preferences according to dopamine receptor affinity of used antipsychotic medications

Experiences with different levels of dopamine receptor affinity contribute to heterogeneity in medication preferences. We did not observe the hypothesized association between the previous usage of high dopamine D _2_ -affinity agents and corresponding reluctance to these side effects including EPSs. A likely explanation is that aversion to side effects are not necessarily related to prior experiences, but rather reflect anticipatory preferences. Another possible explanation is the categorization of specific side effects into broader groups, which may mask important differences in how individual symptoms are experienced. For instance, EPSs were evaluated using elements related to sore muscles, tremors, and akathisia—symptoms that vary in occurrence and severity depending on the specific antipsychotic medication used. 48​ ​49​ Similarly, sexual dysfunction was evaluated by the acceptability of changes in libido and difficulties achieving an orgasm. While relevant, these factors represent only certain aspects of sexual dysfunction and may not fully capture the overall impact on patient experience. 50​ 51​ ​52​

### Strengths and limitations

The major strength of this study is its large sample size, providing robust statistical power and minimizing the risk of type I and type II errors despite multiple comparisons. The easy access to the PAC-index and the privacy experienced increases the plausibility that the results are relevant to a broad population, enhancing the generalizability of the results. Furthermore, the inclusion of a wide range of medications allows for a reflection of real-world treatment preferences, increasing the valuable use for clinicians and external validity.

While this study provides unique information about antipsychotic medication preferences, there are several methodological limitations that should be acknowledged in the interpretation of the results. A key limitation of this study is the potential selection bias inherent to its self-selected, online design. Patients who do not acknowledge their experiences as psychosis or mark them differently may not be included in the study population. This could occur in two ways: first, because they choose not to complete the PACindex, and second, because they report not experiencing psychosis or are unsure having a psychosis. As a result, the representativeness of the sample could be limited. This bias likely favors participants who are higher functioning or more treatment-engaged and may limit the generalizability of the findings, particularly to long-term care settings where patients with more severe or chronic conditions are more common, in whom the awareness of illness is often limited and having a different perspective on symptoms than their healthcare professionals is more common. Another key limitation is the exclusive reliance on self-reported data without clinician verification. As a result, we cannot verify the accuracy or validity of participants’ responses, nor exclude the possibility of repeated entries, leading to the possible response bias. This lack of verification introduces uncertainty regarding the validity and reliability of the data, and unfortunately, this information cannot be retrieved. Another limitation is the ability to evaluate how preferences vary across diverse population groups, due to missing detailed demographic data (e.g. ethnicity or socioeconomic status), a critical gap in research that often overlooks less-represented communities. This omission is particularly relevant given prior findings that patients from non-white ethnic backgrounds report greater dissatisfaction with treatment, 53​ ​54​ and individuals of Asian descent may exhibit differential susceptibility to antipsychotic medications. 55​ ​56​ Furthermore, the lack of detailed clinical data, such as illness duration, treatment phase, symptom severity and current side effects, prevents a detailed understanding of how these factors may shape medication preferences. Factors like illness stage or current symptom burden may influence tolerance for side effects, while the absence of information on current side effects obscures whether preferences reflect lived experiences or hypothetical scenarios. Finally, certain questionnaire items could have been open to multiple interpretations, which could have led to response inconsistencies.

Although a detailed comparison of pharmacological efficacy falls outside the scope of the present study, it should be noted that the largely comparable effectiveness of most antipsychotic medications limits the possibility to distinguish treatment options on therapeutic benefit alone.

### Future directions

The above-mentioned considerations underscore the need for future research to incorporate comprehensive demographic and clinical data to better understand the nuanced relationship between antipsychotic use, symptomatology, and treatment preferences. Additionally, more detailed information could enable more tailored and personalized recommendations. For instance, different medications, each with unique receptor affinities, are associated with varying types and severities of sexual side effects. Understanding these distinctions can guide clinicians in selecting medications that minimize sexual dysfunction, thereby improving treatment adherence and patient outcomes. 46​ ​57​

## Conclusions

Current findings provide a comprehensive overview of the diverse patient preferences regarding the effects and side effects of antipsychotic medications, offering helpful insights to support clinicians in optimizing shared decision making. A key contribution of this study is the identification of differences in preferences between patient groups, highlighting the importance of tailoring treatment to individual needs rather than assuming a one-size-fits-all approach. While this study focused on group-level differences, it is likely that significant variations also exist within these groups, further underscoring the necessity of personalized treatment discussions. Our current study could be a starting point, where our results provide a direction within the clinical setting, from where other preferences can be examined. Ultimately, the current study strengthens the case for a more patient-centered approach in psychiatric care, where treatment decisions are not only guided by clinical evidence but also informed by individual preferences and experiences. Moving forward, integrating these perspectives into practice will be essential for improving outcomes and advancing the quality of mental health care.

## Supplementary Material

Supplementary Material Zusatzmaterial
