# Supplementary material for: Antipsychotic Medication Preferences: A Large-Scale Survey
Source: Pharmacopsychiatry. 2026 Feb 19;59(5):311–9. doi: 10.1055/a-2784-3444 (PMC13569402; doi:10.1055/a-2784-3444)
Supplement: Supplementary file 1 [file 10-1055-a-2784-3444_29442238.pdf]

Supplementary Figure titles and legends

Supplementary Figure 1. Preferences on medication intake, in % Yes/No

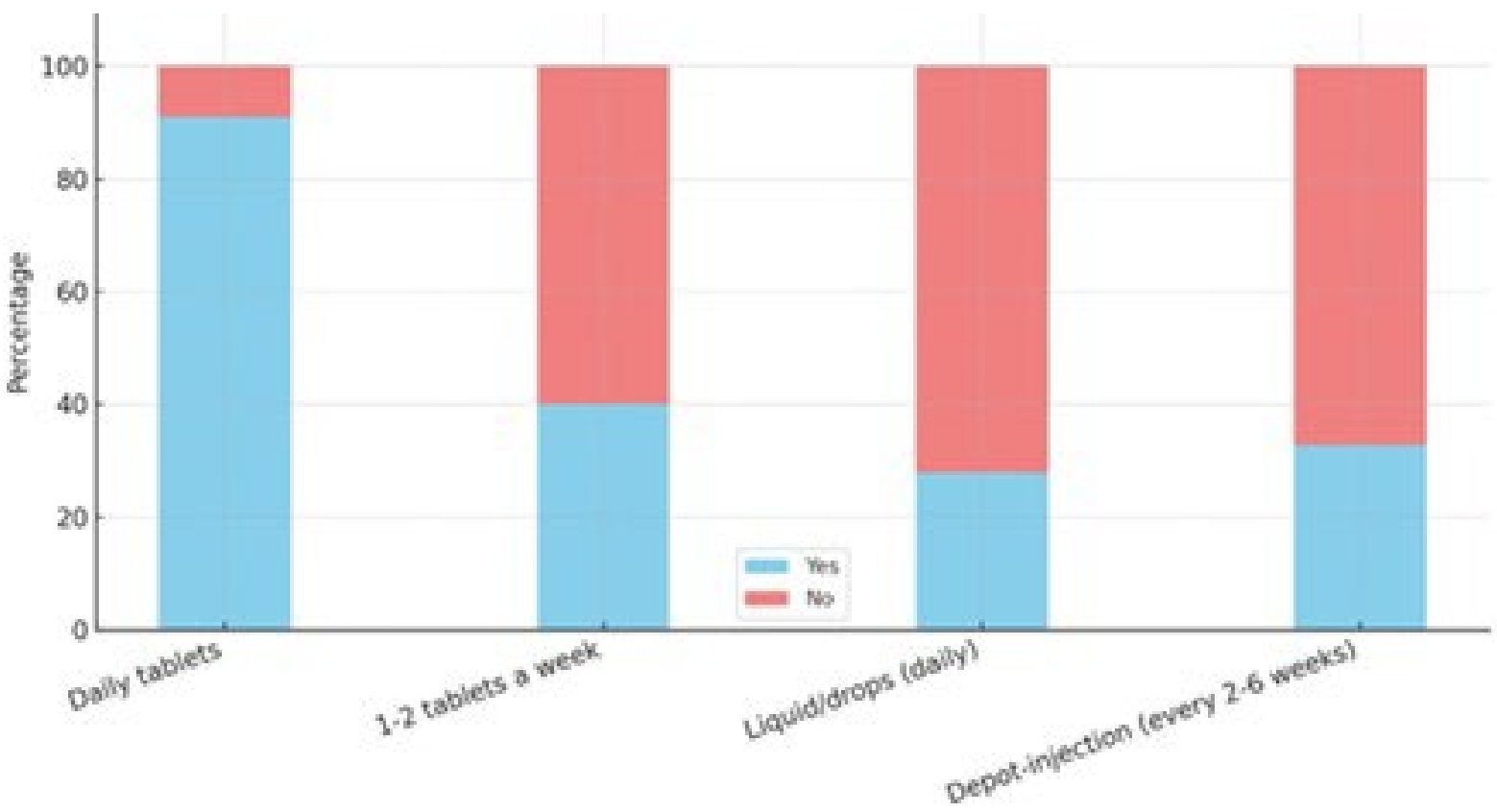

Supplementary Figure 2. Preferences on effectiveness, in mean Likert scores (past D_2 medication user)

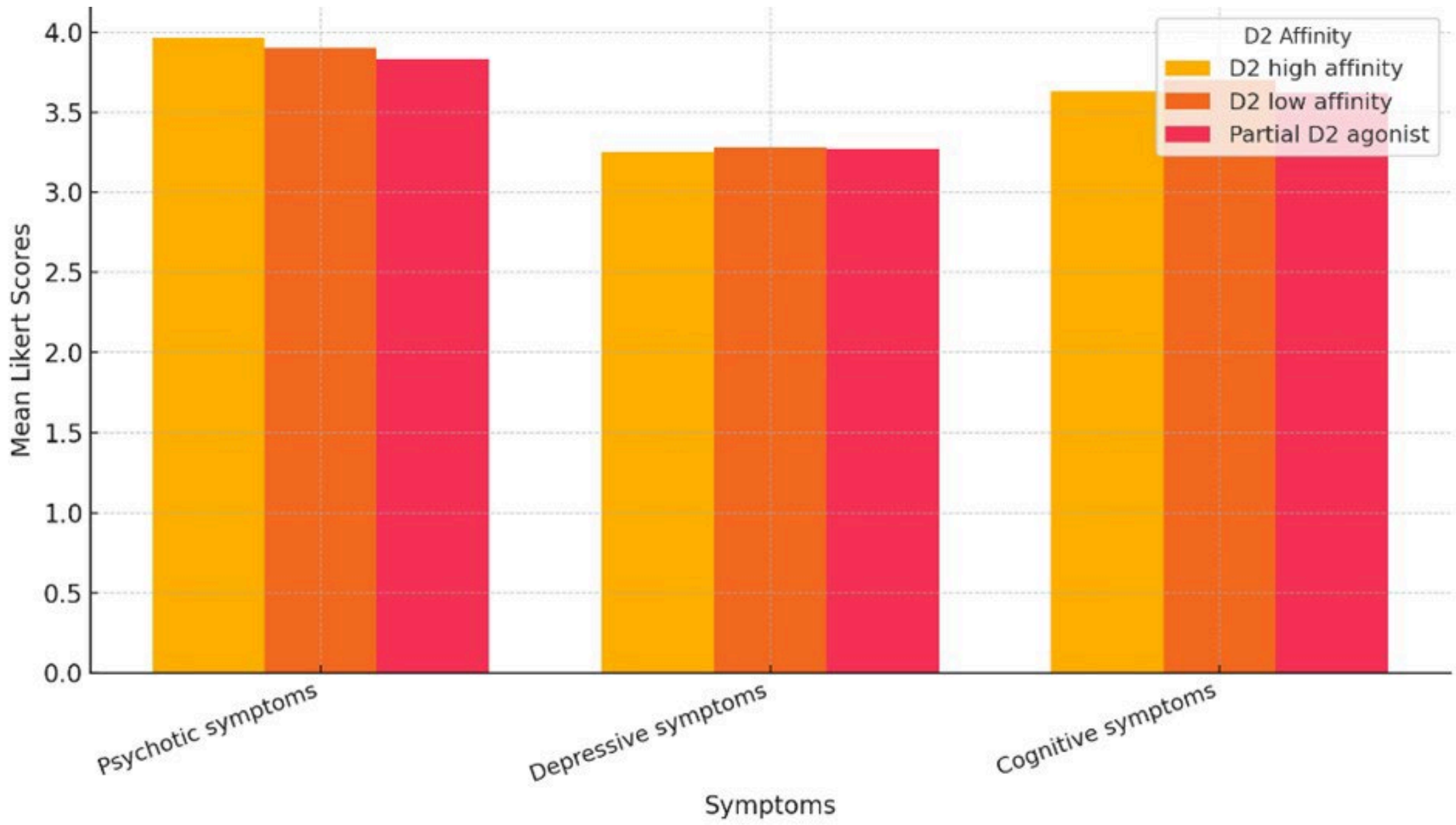

Legend:
Likert score 1 = “Very unimportant”, score 2 = “Unimportant”, score 3 = “Neutral”, score 4 = “Important”, score 5 = “Very important”
Abbreviations: D_2 = dopamine D_2 receptor

Supplementary Figure 3. Preferences on side effects, in mean Likert scores (past D_2 medication user)

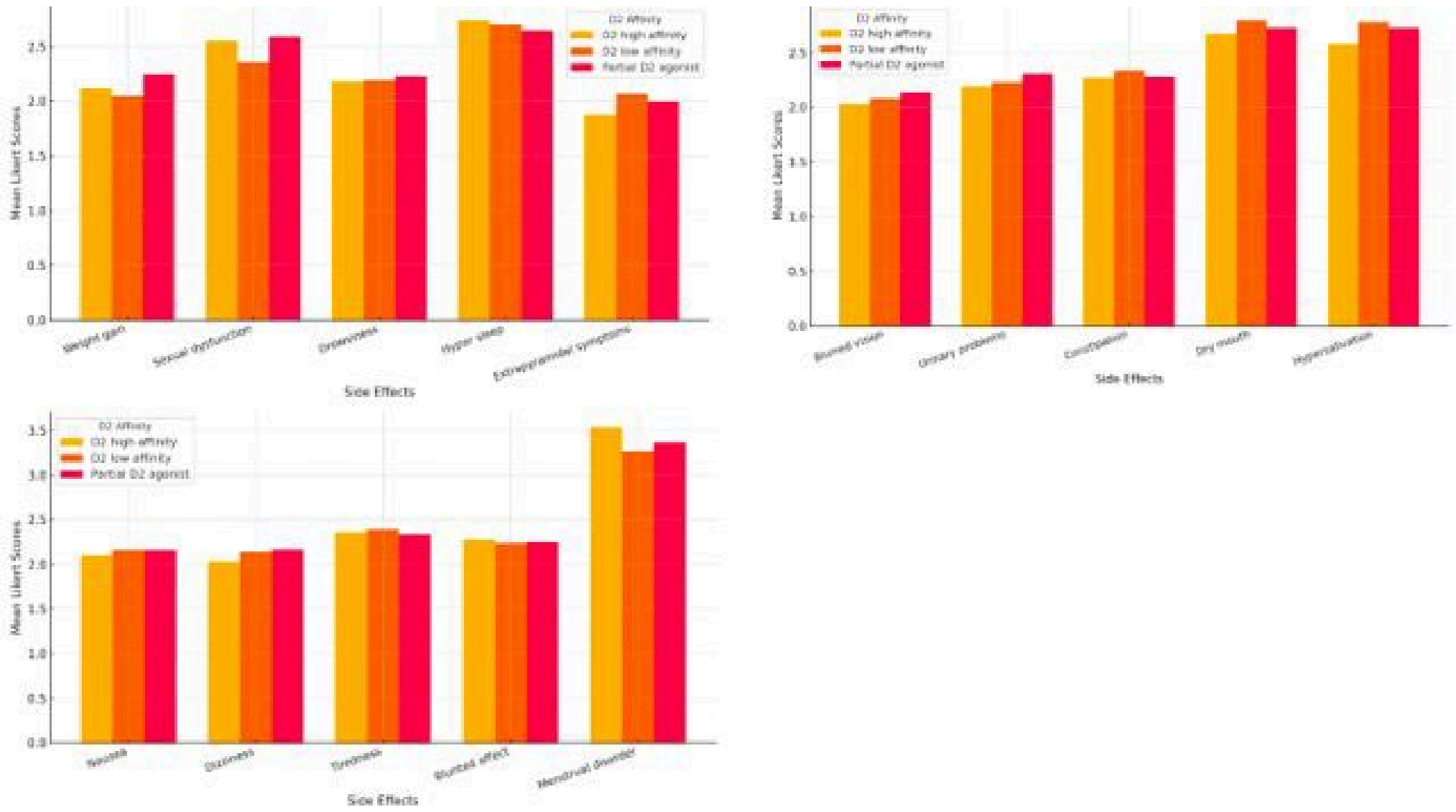

Legend:
Likert score 1 = “Very unacceptable”, score 2 = “Unacceptable”, score 3 = “Neutral”, score 4 = “Acceptable”, score 5 = “Very acceptable”
Abbreviations: D_2 = dopamine D_2 receptor

Supplementary Figure 4. Preferences on effectiveness, in mean Likert scores (current D_2 medication user)

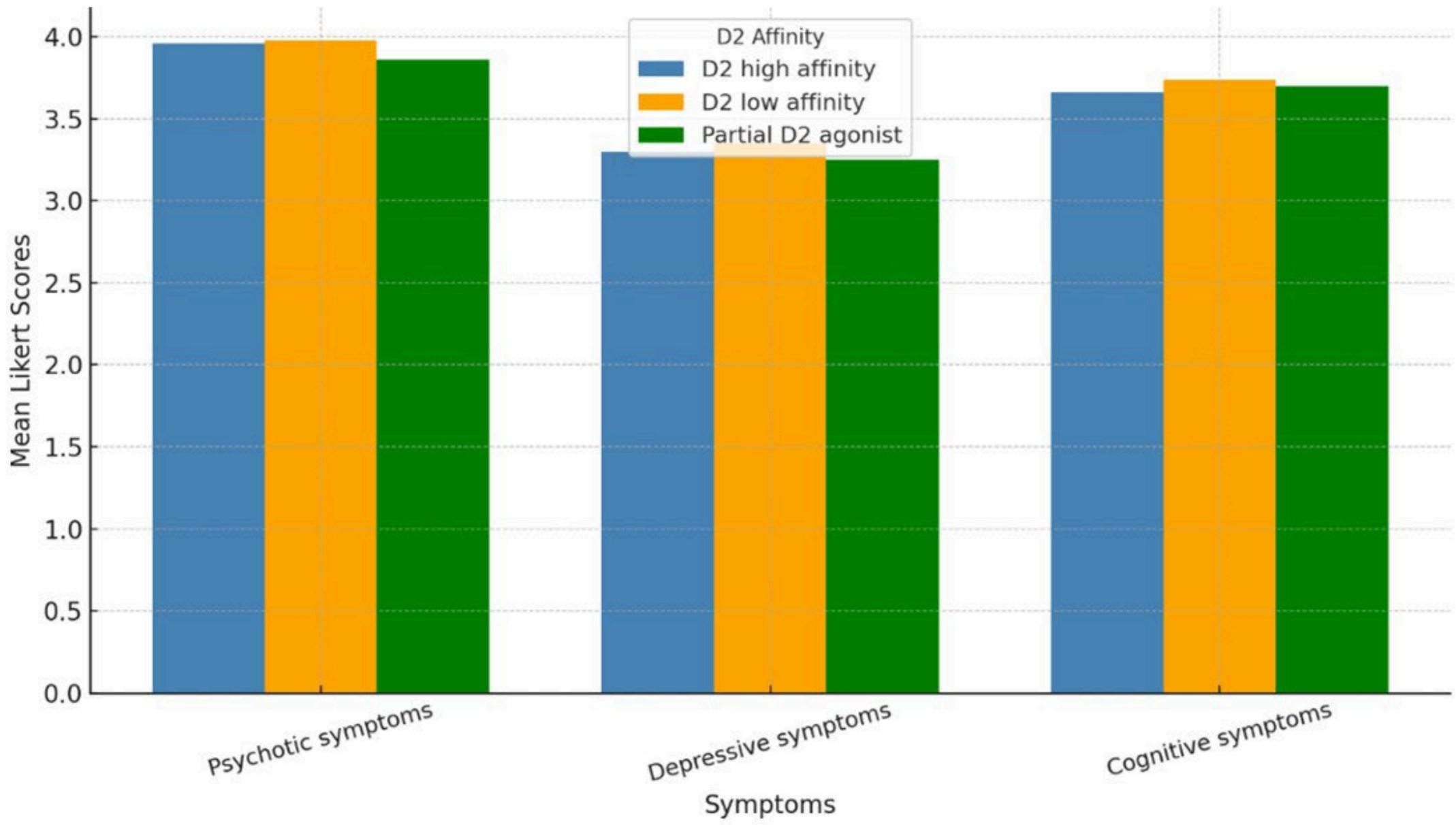

Legend:
Likert score 1 = “Very unimportant”, score 2 = “Unimportant”, score 3 = “Neutral”, score 4 = “Important”, score 5 = “Very important”
Abbreviations: D_2 = dopamine D_2 receptor

Supplementary Figure 5. Preferences on side effects, in mean Likert scores (current D_2 medication user)

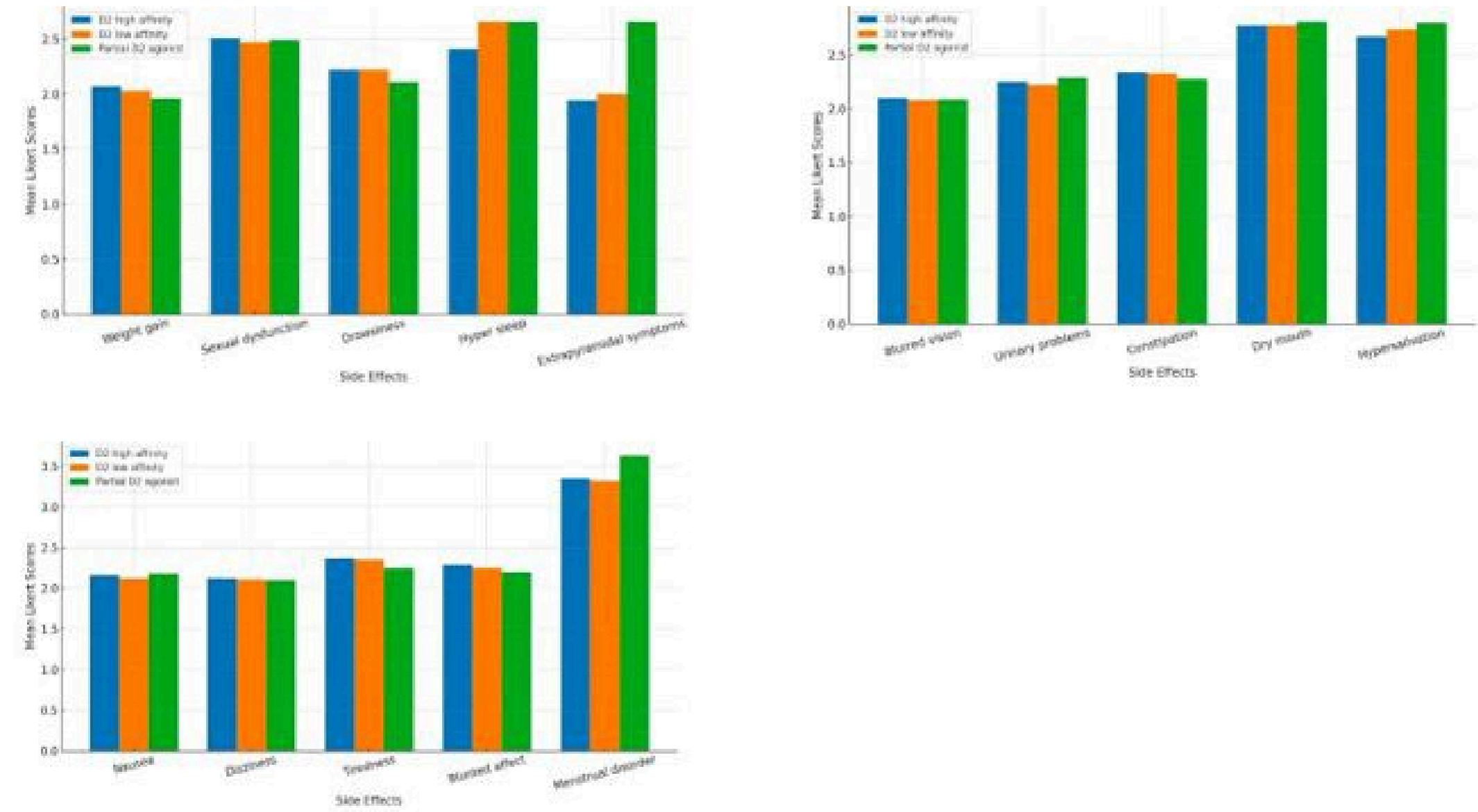

Legend:
Likert score 1 = “Very unacceptable”, score 2 = “Unacceptable”, score 3 = “Neutral”, score 4 = “Acceptable”, score 5 = “Very acceptable”
Abbreviations: D_2 = dopamine D_2 receptor

Supplementary Table 1 – 29

Supplementary Table 1: Patients divided in five age categories in total number of respondents and percentages

	n	%	Valid %
**<20 years**	323	3.7	3.9
**20-35 years**	3767	43.1	45.5
**36-50 years**	2468	28.2	29.7
**51-65 years**	1339	15.3	16.1
**65> years**	400	4.6	4.8
**Total**	8297	94.9	100
**Missing**	446	5.1	

Supplementary Table 2: Categorization of different types of antipsychotic medications

high-affinity D_2 antagonists	Flupentixol, haloperidol, pimozide, risperidone, zuclopenthixol, sulpride, paliperidone, penfluridol, amisulpride, pipamperone and lurasidone
low-affinity D_2 antagonists	Clozapine, olanzapine, quetiapine
partial D_2 agonists	Aripiprazole

Abbreviations: D_2 = dopamine 2 receptor

Supplementary Table 3. Descriptives of past and current use of D2 affinity of respondents with psychosis, n (valid %)

	n	%
**Past users**		
**high-affinity D_2 antagonists**	4.791	54.8%
**low-affinity D_2 antagonists**	5.263	60.2%
**partial D_2 agonists**	2.261	25.9%
**Current users**		
**high-affinity D_2 antagonists**	2.820	32.3%
**low-affinity D_2 antagonists**	3.225	36.9%
**partial D_2 agonists**	959	11.0%

Additional: 3.798 only used AP out of 1 group (43.4%), 2.448 (28.0%) out of 2 groups and 1.207 (13.8%) used antipsychotics out of all three groups
6.067 use AP out of 1 group (69.4%), 452 (5.2%) out of 2 groups and 11 (0.1%) used antipsychotics out of all 3 groups.
Abbreviations: D_2 = dopamine D_2 receptor, AP = antipsychotic medication

Supplementary Table 4. Preferences on effectiveness by respondents with psychosis, n (valid %)

	Psychotic symptoms	Depressive symptoms	Cognitive symptoms
**"Very important"**	2.416 (30.8%)	1.358 (18.2%)	1.911 (25.1%)
**"Important"**	3.504 (44.6%)	2.334 (31.3%)	2.804 (36.8%)
**"Neutral"**	1.280 (16.3%)	2.025 (27.1%)	2.019 (26.5%)
**"Unimportant"**	280 (3.6%)	864 (11.6%)	504 (6.6%)
**"Very unimportant"**	368 (4.7%)	884 (11.8%)	386 (5.1%)
**Missing Data**	895 (10.2%)	1.278 (14.6%)	1.119 (12.8%)

Supplementary Table 5. Wilcoxon Signed Ranks test of differences on effectiveness

	**Depressive symptoms**<br>mean = 3.32 (SD = 1.24)	**Cognitive symptoms**<br>mean = 3.70 (SD = 1.07)
**Psychotic symptoms**<br>mean = 3.93 (SD = 1.02)	$p < 0.001^{*a}$	$p < 0.001^{*a}$
**Depressive symptoms**<br>mean = 3.32 (SD = 1.24)	X	$p < 0.001^{*b}$

*$p < 0.017$ (Bonferroni 0.05/3)

[a]Preferable effect on psychotic symptoms

[b]Preferable effect on cognitive symptoms

Abbreviations: SD = standard deviation

Supplementary Table 6. Preferences on side effects by respondents with psychosis, n (valid %)

	Very unacceptable	Unacceptable	Neutral	Acceptable	Very acceptable	Missing Data
**Weight gain**	3.034 (41.3%)	1.964 (26.7%)	1.448 (19.7%)	712 (9.7%)	188 (2.6%)	1.397 (16.0%)
**Sexual dysfunction**	1.970 (27.3%)	1.663 (23.0%)	2.207 (30.5%)	887 (12.3%)	502 (6.9%)	1.514 (17.3%)
**Drowsiness**	2.102 (28.8%)	2.619 (35.9%)	1.690 (23.2%)	716 (9.8%)	169 (2.3%)	1.447 (16.6%)
**Hyper sleep**	1.219 (16.8%)	1.861 (25.7%)	2.255 (31.1%)	1.544 (21.3%)	364 (5.0%)	1.500 (17.2%)
**Extrapyramidal symptoms**	2.692 (37.2%)	2.623 (36.0%)	1.469 (20.3%)	372 (5.1%)	8 (1.2%)	1.501 (17.2%)
**Blurred vision**	2.181 (30.5%)	2.784 (38.9%)	1.734 (24.3%)	350 (4.9%)	101 (1.4%)	1.593 (18.2%)
**Urinary problems**	1.796 (25.3%)	2.518 (35.5%)	2.143 (30.2%)	530 (7.5%)	112 (1.6%)	1.644 (18.8%)
**Constipation**	1.612 (22.7%)	2.351 (33.1%)	2.450 (34.5%)	596 (8.4%)	97 (1.4%)	1.637 (18.7%)
**Dry mouth**	841 (12.0%)	1.631 (23.2%)	2.082 (43.8%)	1.257 (17.9%)	222 (3.2%)	1.710 (19.6%)
**Hypersalivation**	984 (14.0%)	1.709 (24.3%)	2.918 (41.4%)	1.190 (16.9%)	239 (3.4%)	1.703 (19.5%)
**Nausea**	1.816 (25.9%)	2.898 (41.3%)	1.868 (26.6%)	338 (4.8%)	96 (1.4%)	1.727 (19.8%)
**Dizziness**	1.906 (27.2%)	2.923 (41.7%)	1.747 (24.9%)	359 *5.1%)	72 (1.0%)	1.736 (19.9%)
**Tiredness**	1.587 (22.5%)	2.356 (33.4%)	2.186 (31.0%)	789 (11.2%)	131 (1.9%)	1.694 (19.4%)
**Blunted affect**	2.008 (28.4%)	2.144 (30.4%)	2.135 (30.2%)	622 (8.8%)	149 (2.1%)	1.685 (19.3%)
**Menstrual disorder**	307 (11.3%)	254 (9.3%)	785 (28.9%)	675 (24.8%)	697 (25.6%)	6.025 (68.9%)

Supplementary Table 7. Wilcoxon Signed Ranks test of differences on tolerability
Friedman test on tolerability, *p* < .001

Side effect	Successive side effect	*p*
**Extrapyramidal symptoms**<br>mean 1.97 (SD = 0.94)	**Weight gain**<br>mean = 2.05 (SD = 1.11)	< .001*
**Weight gain**<br>mean = 2.05 (SD = 1.11)	**Blurred vision**<br>mean = 2.08 (SD = 0.93)	.108
**Weight gain**<br>mean = 2.05 (SD = 1.11)	**Dizziness**<br>mean = 2.11 (SD = 0.90)	< .001*
**Blurred vision**<br>mean = 2.08 (SD = 0.93)	**Dizziness**<br>mean = 2.11 (SD = 0.90)	.001*
**Dizziness**<br>mean = 2.11 (SD = 0.90)	**Nausea**<br>mean = 2.14 (SD = 0.91)	.001*
**Nausea**<br>mean = 2.14 (SD = 0.91)	**Drowsiness**<br>mean = 2.21 (SD = 1.04)	<.001*
**Drowsiness**<br>mean = 2.21 (SD = 1.04)	**Urinary problems**<br>mean = 2.25 (SD = 0.97)	.004
**Drowsiness**<br>mean = 2.21 (SD = 1.04)	**Blunted affect**<br>mean = 2.26 (SD = 1.03)	<.001*
**Urinary problems**<br>mean = 2.25 (SD = 0.97)	**Blunted affect**<br>mean = 2.26 (SD = 1.03)	.442
**Urinary problems**<br>mean = 2.25 (SD = 0.97)	**Constipation**<br>mean = 2.33 (SD = 0.96)	< .001*
**Blunted affect**<br>mean = 2.26 (SD = 1.03)	**Constipation**<br>mean = 2.33 (SD = 0.96)	<.001*
**Constipation**<br>mean = 2.33 (SD = 0.96)	**Tiredness**<br>mean = 2.36 (SD = 1.01	.005
**Constipation**<br>mean = 2.33 (SD = 0.96)	**Sexual dysfunction**<br>mean = 2.49 (SD = 1.21)	<.001*
**Tiredness**<br>mean = 2.36 (SD = 1.01)	**Sexual dysfunction**<br>mean = 2.49 (SD = 1.21)	< .001*
**Sexual dysfunction**<br>mean = 2.49 (SD = 1.21)	**Hypersalivation**<br>mean = 2.71 (SD = 1.01)	<.001*
**Hypersalivation**<br>mean = 2.71 (SD = 1.01)	**Hyper sleep**<br>mean 2.72 (SD – 1.13)	.739
**Hypersalivation**<br>mean = 2.71 (SD = 1.01)	**Dry mouth**<br>mean = 2.77 (SD = 0.98)	<.001*
**Hyper sleep**<br>mean 2.72 (SD – 1.13)	**Dry mouth**<br>mean = 2.77 (SD = 0.98)	.001*
**Dry mouth**<br>mean = 2.77 (SD = 0.98)	**Menstrual disorder**<br>mean 3.44 (SD = 1.28)	<.001*

Higher scores indicate higher unacceptability
*p < .0025 (Bonferroni 0.05/20 = 0.0025)
Abbreviations: SD = standard deviation

Supplementary Table 8. Preferences on medication intake by respondents with psychosis, n (valid %)

	Daily tablets	1-2 tablets a week	liquid/drops (daily)	depot-injection (every 2-6 weeks)
"Yes"	6.997 (91.0%)	3.082 (40.1%)	2.144 (27.9%)	2.518 (32.7%)
"No"	696 (9.0%)	4.611 (59.9%)	5.549 (72.1%)	5.175 (67.3%)
Missing Data	1.050 (12.0%)	1.050 (12.0%)	1.050 (12.0%)	1.050 (12.0%)

Supplementary Table 9. Wilcoxon Signed Ranks test of differences on mediation intake

	**Daily tablets** mean = 1.09 (SD = 0.28)	**1-2 tablets a week** mean = 1.60 (SD = 0.49)	**Liquid/drops (daily)** mean = 1.72 (SD = 0.45)
**1-2 tablets a week** mean = 1.60 (SD = 0.49)	$p = 0.000$*[a]	X	$p < 0.001$[b]
**Liquid/drops (daily)** mean = 1.72 (SD = 0.45)	$p = 0.000$*[a]	$p < 0.001$*[b]	X
**Depot-injection (every 2-6 weeks)** mean = 1.67 (SD = 0.47)	$p = 0.000$*[a]	$p < 0.001$*[b]	$p < 0.001$[c]

*$p < .008$ (Bonferroni 0.05/6)
[a]Preferable intake on daily tablets
[b]Preferable intake on 1-2 tablets a week
[c]Preferable intake on depot-injection
Abbreviations: SD = standard deviation

Supplementary Table 10. Mann-Whitney U tests for sex differences in preferences on effectiveness by respondents with psychosis, in mean rank and Likert scores (p-value)

	Man	Woman	p-value
**Psychotic symptoms**	3850,63<br>3,90	3996,15<br>3,99	.003*[a]
**Depressive symptoms**	3603,26<br>3,26	3881,01<br>3,42	<.001*[a]
**Cognitive symptoms**	3756,57<br>3,68	3856,62<br>3,74	.041

*$p < .05$
[a]Women find it more important than men

Supplementary Table 11. Mann-Whitney U tests for sex differences in preferences on side-effects by respondents with psychosis, in mean rank and Likert scores (p-value).

	Man	Woman	p-value
**Extrapyramidal symptoms**	3673,16<br>2,00	3516,19<br>1,93	<.001*[a]
**Weight gain**	4018,12<br>2,24	3145,87<br>1,78	<.001*[a]
**Blurred vision**	3607,03<br>2,10	3499,07<br>2,04	.021
**Dizziness**	3538,61<br>2,13	3423,72<br>2,08	.013
**Nausea**	3520,26<br>2,15	3460,60<br>2,13	.198
**Drowsiness**	3664,18<br>2,23	3592,28<br>2,18	.133
**Urinary problems**	3527,87<br>2,24	3548,72<br>2,25	.658
**Blunted affect**	3547,53<br>2,27	3469,97<br>2,24	.100
**Constipation**	3546,55<br>2,33	3532,01<br>2,32	.757
**Tiredness**	3520,09<br>2,37	3499,32<br>2,36	.659
**Sexual dysfunction**	3283,31<br>2,29	4063,29<br>2,77	<.001*[b]
**Hypersalivation**	3560,13<br>2,74	3431,99<br>2,67	.006
**Hyper sleep**	3578,18<br>2,70	3651,56<br>2,75	.128
**Dry mouth**	3518,85<br>2,78	3481,86<br>2,76	.426
**Menstrual disorder**	0	1359,50<br>3,44	[c]

*$p < .05$
[a]Women find it more unacceptable
[b]Men find it more unacceptable
[c]Mann-Whitney Test cannot be performed on empty groups

Supplementary Table 12. Age differences in preferences on effectiveness by respondents with psychosis, in mean Likert scores

	**<20**	**20-35**	**36-50**	**51-65**	**>65**
**Psychotic symptoms**	4.06	3.91	3.97	3.99	3.94
**Depressive symptoms**	3.38	3.36	3.30	3.29	3.30
**Cognitive symptoms**	3.79	3.77	3.65	3.65	3.57

Higher scores indicate higher importance

Supplementary Table 13. Kruskal-Wallis Test for age differences in preferences on effectiveness by respondents with psychosis, in Kruskal-Wallis H value, (p-value).

	Kruskal-Wallis H	p-value
**Psychotic symptoms**	9,359	0.053
**Depressive symptoms**	6,476	0.166
**Cognitive symptoms**	40,366	<.001*

Supplementary Table 14. Post-hoc Mann-Whitney U test for age differences in preferences on effectiveness of cognitive symptoms, p-value

	20-35	36-50	51-65	>65
**<20**	.949	.016	.024	.004*[a]
**20-35**	X	<.001*[b]	<.001*[b]	<.001*[b]
**36-50**	<.001*[b]	X	.831	.159
**51-65**	<.001*[b]	.831	X	.131
**>65**	<.001*[b]	.159	.131	X

*$p < .05$

[a]<20 years of age find it more important

[b]20-35 find it more important

Supplementary Table 15. Age differences in preferences on side-effects by respondents with psychosis, in mean Likert scores

	<20	20-35	36-50	51-65	>65
**Extrapyramidal symptoms**	2.09	1.99	1.98	1.88	1.86
**Weight gain**	2.00	2.07	1.98	2.04	2.54
**Blurred vision**	2.09	2.06	2.09	2.04	2.21
**Dizziness**	2.14	2.12	2.15	2.02	2.03
**Nausea**	2.15	2.12	2.18	2.06	2.28
**Drowsiness**	2.21	2.17	2.26	2.21	2.25
**Urinary problems**	2.26	2.24	2.24	2.22	2.27
**Blunted affect**	2.21	2.17	2.29	2.33	2.64
**Constipation**	2.32	2.32	2.35	2.35	2.41
**Tiredness**	2.45	2.30	2.41	2.36	2.57
**Sexual dysfunction**	2.61	2.35	2.39	2.74	3.51
**Hypersalivation**	2.86	2.75	2.69	2.59	2.70
**Hyper sleep**	2.71	2.63	2.74	2.88	2.90
**Dry mouth**	2.82	2.80	2.77	2.66	2.80
**Menstrual disorder**	3.39	3.18	3.34	3.98	4.36

Higher scores indicate higher acceptability

Supplementary Table 16. Kruskal-Wallis Test for age differences in preferences on side-effects by respondents with psychosis, in Kruskal-Wallis H value, (p-value).

	Kruskal-Wallis H	p-value
Extrapyramidal symptoms	19,554	<.001
Weight gain	70,495	<.001
Blurred vision	11,143	.025
Dizziness	14,938	<.001
Nausea	21,961	<.001
Drowsiness	14.633	.006
Urinary problems	0,674	.955
Blunted affect	82,057	<.001
Constipation	7,378	.117
Tiredness	34,023	<.001
Sexual dysfunction	314,106	<.001
Hypersalivation	26,525	<.001
Hyper sleep	52,028	<.001
Dry mouth	20,534	<.001
Menstrual disorder	215,580	<.001

Supplementary Table 17. Post-hoc Mann-Whitney U test for age differences in preferences on side effects, p-value

	20-35	36-50	51-65	>65
Extrapyramidal symptoms				
<20	.123	.100	.003*[a]	.002*[a]
20-35	X	.785	.003*[b]	.007*[b]
36-50	.785	X	.008*[c]	.011*[c]
51-65	.003*[b]	.008*[c]	X	.332
>65	.007*[b]	.011*[c]	.332	X
Weight gain				
<20	.215	.703	.231	<.001*[e]
20-35	X	.037*[b]	.997	<.001*[e]
36-50	.037*[b]	X	.091	<.001*[e]
51-65	.997	.091	X	<.001*[e]
>65	<.001*[e]	<.001*[e]	<.001*[e]	X
Dizziness				
<20	.795	.650	.135	.272
20-35	X	.098	.013*[b]	.199
36-50	.098	X	<.001*[c]	.037*[c]
51-65	.013*[b]	<.001*[c]	X	.871
>65	.199	.037*[c]	.871	X
Nausea				
<20	.736	.482	.259	.043*[e]

**20-35**	X	.018*c	.116	.001*e
**36-50**	.018*c	X	<.001*c	.043*e
**51-65**	.116	<.001*c	X	<.001*e
**>65**	.001*e	.043*e	<.001*e	X
**Blunted affect**				
**<20**	.532	.159	.031*d	<.001*e
**20-35**	X	<.001*c	<.001*d	<.001*e
**36-50**	<.001*c	X	.204	<.001*e
**51-65**	<.001*d	.204	X	<.001*e
**>65**	<.001*e	<.001*e	<.001*e	X
**Tiredness**				
**<20**	.056	.706	.352	.110
**20-35**	X	<.001*c	.033*d	<.001*e
**36-50**	<.001*c	X	.250	.003*e
**51-65**	.033*d	.250	X	<.001*e
**>65**	<.001*e	.003*e	<.001*e	X
**Sexual dysfunction**				
**<20**	<.001*a	.008*a	.075	<.001*e
**20-35**	X	.118	<.001*b	<.001*e
**36-50**	.118	X	<.001*d	<.001e
**51-65**	<.001*b	<.001*d	X	<.001*e
**>65**	<.001*e	<.001e	<.001*e	X
**Hypersalivation**				
**<20**	.091	.011	<.001*a	.037*a
**20-35**	X	.063	<.001*b	.343
**36-50**	.063	X	.006*c	.985
**51-65**	<.001*b	.006*c	X	.084
**>65**	.343	.985	.084	X
**Hyper sleep**				
**<20**	.294	.577	0.028*d	.041*e
**20-35**	X	<.001*c	<.001*d	<.001*e
**36-50**	<.001*c	X	.002*d	.027*e
**51-65**	<.001*d	.002*d	X	.784
**>65**	<.001*e	.027*e	.784	X
**Dry mouth**				
**<20**	.577	.278	.004*a	.453
**20-35**	X	.237	<.001*b	.834
**36-50**	.237	X	.002*c	.662
**51-65**	<.001*b	.002*c	X	.013*e

**>65**	.834	.662	.013*[e]	X
**Menstrual disorder**				
**<20**	.159	.793	<.001*[d]	<.001*[e]
**20-35**	X	.011*[c]	<.001*[d]	<.001*[e]
**36-50**	.011*[c]	X	<.001*[d]	<.001*[e]
**51-65**	<.001*[d]	<.001*[d]	X	<.001*[e]
**>65**	<.001*[e]	<.001*[e]	<.001*[e]	X

*$p < .05$
[a]<20 more acceptable
[b]20-35 more acceptable
[c]36-50 more acceptable
[d]51-65 more acceptable
[e]>65 more acceptable

Supplementary Table 18. Past D_2 affinity medication user differences in preferences on effectiveness by respondents with psychosis, in mean Likert scores

	**high-affinity D_2 antagonists**	**low-affinity D_2 antagonists**	**partial D_2 agonists**
**Psychotic symptoms**	3.96	3.90	3.83
**Depressive symptoms**	3.25	3.28	3.27
**Cognitive symptoms**	3.63	3.70	3.62

Higher scores indicate higher importance
Abbreviations: D_2 = dopamine D_2 receptor

Supplementary Table 19. Kruskal-Wallis Test for past D_2 affinity medication user differences in preferences on effectiveness by respondents with psychosis and using pastally one type of antipsychotic medication, in Kruskal-Wallis H value, (p-value).

	**Kruskal-Wallis H**	**p-value**
**Psychotic symptoms**	2,967	.227
**Depressive symptoms**	0,677	0.713
**Cognitive symptoms**	6,089	.048*

*$p < 0.05$
Abbreviations: D_2 = dopamine D_2 receptor

Supplementary Table 20. Post-hoc Mann-Whitney U test for past D_2 affinity medication differences in preferences on effectiveness of cognitive symptoms, p-value

	**partial D_2 agonists**	**low-affinity D_2 antagonists**
**high-affinity D_2 antagonists**	.952	.016*[a]
**low-affinity D_2 antagonists**	.303	X

*$p<.01667$ (0.05/3)
[a]In favor of low D_2 affinity
Abbreviations: D_2 = dopamine D_2 receptor

Supplementary Table 21. Past D_2 affinity medication user differences in preferences on side effects by respondents with psychosis, in mean Likert scores

	high-affinity D_2 antagonists	low-affinity D_2 antagonists	partial D_2 agonists
**Extrapyramidal symptoms**	1.88	2.07	2.00
**Weight gain**	2.12	2.05	2.25
**Blurred vision**	2.03	2.08	2.14
**Dizziness**	2.03	2.14	2.17
**Nausea**	2.10	2.16	2.16
**Drowsiness**	2.18	2.19	2.23
**Urinary problems**	2.19	2.23	2.31
**Blunted affect**	2.28	2.24	2.25
**Constipation**	2.27	2.34	2.28
**Tiredness**	2.36	2.39	2.34
**Sexual dysfunction**	2.55	2.36	2.59
**Hypersalivation**	2.58	2.78	2.73
**Hyper sleep**	2.74	2.70	2.65
**Dry mouth**	2.67	2.80	2.73
**Menstrual disorder**	3.53	3.27	3.37

Higher scores indicate higher acceptability
Abbreviations: D_2 = dopamine D_2 receptor

Supplementary Table 22. Kruskal-Wallis Test for past D_2 affinity medication user differences in preferences on side effects by respondents with psychosis and using only one type of antipsychotic medication, in Kruskal-Wallis H value, (p-value).

	Kruskal-Wallis H	p-value
**Extrapyramidal symptoms**	32,812	.102
**Weight gain**	5,712	.058
**Blurred vision**	2,907	.234
**Dizziness**	10,370	.006*
**Nausea**	2,836	.242
**Drowsiness**	0,197	.906
**Urinary problems**	2,578	.276
**Blunted affect**	1,898	.387
**Constipation**	2,471	.291
**Tiredness**	0,579	.752
**Sexual dysfunction**	17,559	<.001*
**Hypersalivation**	26,920	<.001*
**Hyper sleep**	1,730	.421
**Dry mouth**	13,700	.001
**Menstrual disorder**	11,225	.004*

$P < 0.05$
Abbreviations: D_2 = dopamine D_2 receptor

Supplementary Table 23. Post-hoc Mann-Whitney U test for past D_2 affinity medication differences in preferences on side effects, p-value

	partial D_2 agonists	low-affinity D_2 antagonists
**Dizziness**		
high-affinity D_2 antagonists	.101	.002*[a]
low-affinity D_2 antagonists	.857	X
**Sexual dysfunction**		
high-affinity D_2 antagonists	.653	<.001*[b]
low-affinity D_2 antagonists	.018*[c]	X
**Hypersalivation**		
high-affinity D_2 antagonists	.083	<.001*[a]
low-affinity D_2 antagonists	.432	X
**Menstrual disorder**		
high-affinity D_2 antagonists	.352	<.001*[b]
low-affinity D_2 antagonists	.490	X

*p<0.05

[a]Low affinity more acceptable

[b]High affinity more acceptable

[c]Partial agonist more acceptable

Abbreviations: D_2 = dopamine D_2 receptor

Supplementary Table 24. Current D_2 affinity medication user differences in preferences on effectiveness by respondents with psychosis, in mean Likert scores

	high-affinity D_2 antagonists	low-affinity D_2 antagonists	partial D_2 agonists
**Psychotic symptoms**	3.96	3.98	3.86
**Depressive symptoms**	3.30	3.35	3.25
**Cognitive symptoms**	3.66	3.74	3.70

Higher scores indicate higher importance

Abbreviations: D_2 = dopamine D_2 receptor

Supplementary Table 25. Kruskal-Wallis Test for current D_2 affinity medication user differences in preferences on effectiveness by respondents with psychosis and using pastally one type of antipsychotic medication, in Kruskal-Wallis H value, (p-value).

	Kruskal-Wallis H	p-value
**Psychotic symptoms**	9,159	.010*
**Depressive symptoms**	4,103	.129
**Cognitive symptoms**	9,484	.009*

Abbreviations: D_2 = dopamine D_2 receptor

Supplementary Table 26. Post-hoc Mann-Whitney U test for current D_2 affinity medication differences in preferences on effectiveness, p-value

	**partial D_2 agonists**	**low-affinity D_2 antagonists**
**Psychotic symptoms**		
high-affinity D_2 antagonists	.009*[b]	.582
low-affinity D_2 antagonists	.003*[a]	X
**Cognitive symptoms**		
high-affinity D_2 antagonists	.166	.002*[a]
low-affinity D_2 antagonists	.524	X

*p<.01667 (0.05/3)

[a]In favor of low affinity

[b]In favor of high affinity

Abbreviations: D_2 = dopamine D_2 receptor

Supplementary Table 27. Current D_2 affinity medication user differences in preferences on side effects by respondents with psychosis, in mean Likert scores

	**high-affinity D_2 antagonists**	**low-affinity D_2 antagonists**	**partial D_2 agonists**
**Extrapyramidal symptoms**	1.94	2.00	2.66
**Weight gain**	2.07	2.03	1.96
**Blurred vision**	2.10	2.08	2.09
**Dizziness**	2.12	2.11	2.10
**Nausea**	2.16	2.12	2.18
**Drowsiness**	2.22	2.22	2.11
**Urinary problems**	2.25	2.22	2.29
**Blunted affect**	2.29	2.25	2.20
**Constipation**	2.34	2.33	2.28
**Tiredness**	2.37	2.35	2.25
**Sexual dysfunction**	2.51	2.47	2.49
**Hypersalivation**	2.67	2.74	2.80
**Hyper sleep**	2.41	2.66	2.66
**Dry mouth**	2.77	2.77	2.81
**Menstrual disorder**	3.35	3.32	3.63

Higher scores indicate higher acceptance

Abbreviations: D_2 = dopamine D_2 receptor

Supplementary Table 28. Kruskal-Wallis Test for current D_2 affinity medication user differences in preferences on side-effects by respondents with psychosis and using currently one type of antipsychotic medication, in Kruskal-Wallis H value, (p-value).

	Kruskal-Wallis H	p-value
Extrapyramidal symptoms	6,682	.017*
Weight gain	6,614	.037*
Blurred vision	1,951	.377
Dizziness	0.211	.900
Nausea	2,980	.225
Drowsiness	7,776	.020*
Urinary problems	2,483	.289
Blunted affect	4,443	.108
Constipation	1,832	.400
Tiredness	8,646	.013*
Sexual dysfunction	0,313	.855
Hypersalivation	10,521	.005*
Hyper sleep	7,524	.023*
Dry mouth	1,238	.539
Menstrual disorder	13,315	.001*

Abbreviations: D_2 = dopamine D_2 receptor

Supplementary Table 29. Post-hoc Mann-Whitney U test for current D_2 affinity medication differences in preferences on side-effects, p-value

	partial D_2 agonists	low-affinity D_2 antagonists
**Extrapyramidal symptoms**		
high-affinity D_2 antagonists	.886	.017*
low-affinity D_2 antagonists	.086	X
**Weight gain**		
high-affinity D_2 antagonists	.013*[b]	.134
low-affinity D_2 antagonists	.131	X
**Drowsiness**		
high-affinity D_2 antagonists	.007*[b]	.775
low-affinity D_2 antagonists	.011*[a]	X
**Tiredness**		
high-affinity D_2 antagonists	.004*[b]	.230
low-affinity D_2 antagonists	.028*[a]	X
**Hypersalivation**		
high-affinity D_2 antagonists	.004*[c]	.016*[a]
low-affinity D_2 antagonists	.199	X
**Hyper sleep**		
high-affinity D_2 antagonists	.096	.008*[b]
low-affinity D_2 antagonists	.974	X
**Menstrual disorder**		
high-affinity D_2 antagonists	.002*[c]	.634
low-affinity D_2 antagonists	<.001*[c]	X

*$p<0.05$

[a]Low affinity more acceptable

[b]High affinity more acceptable

[c]Partial D2 more acceptable

Abbreviations: D_2 = dopamine D_2 receptor

Supplements to 'Antipsychotic Medication Preferences: a Large-Scale Survey'

Babet N. Wezenberg, J. Bouws, M. van der Pluijm, M. de Koning, M. Kikker, M. Blankers[2], I. Sommer, F. van Dijk, L. de Haan

2. Methods

2.3 Setting, Data Collection and Assessments

Supplement, method 1. Full decision aid description
Decision aid link**:** **www.**pakwijzer.nl

Decision aid questionnaire (original in Dutch, translated)
1. Basic questions
Wat is your age? ___

Wat is your gender? □ male □ female

Did you ever have a psychosis? □ yes □ no □ don't know

Which antipsychotic medication did you use in the past? (multiple answers possible)
□ none □ Perphenazine □ Penfluridol □ Olanzapine □ Zuclopentixol □ Pipamperon □ Clozapine □ Pimozide □ Flupentixole □ Quetiapine □ Aripiprazole □ Sulpride □ Risperidone □ Lurasidone □ Amisulpride □ Haloperidol □ Other □ Don't know

Which antipsychotic medication do you use now? (multiple answers possible)
□ none □ Perphenazine □ Penfluridol □ Olanzapine □ Zuclopentixol □ Pipamperon □ Clozapine □ Pimozide □ Flupentixole □ Quetiapine □ Aripiprazole □ Sulpride □ Risperidone □ Lurasidone □ Amisulpride □ Haloperidol □ Other □ Don't know

2. Effects
How important is it that your antipsychotic medication …?
… Works as strongly as possible against psychosis
… Treats your depressive symptoms as strongly as possible
… Improves your memory and concentration problems
Answer: □ no answer □ very unimportant □ unimportant □ neutral □ important □ very important

3. Side effects
How acceptable is it to you that because of your antipsychotic medication you …?
… will gain weight
… are less interested in sex or have difficulty getting an orgasm
… get more drowsy
… sleep more or have difficulty waking up
… get stiff joints, get a tremor or if you make more restless movements
… get blurred vision
… have difficulty urinating
… get constipated
… have dry mouth
… hypersalivate

… get nausea
… experience dizziness
… get more tired easily
… get a blunted affect or be less creative
… menstruate less
Answer: □ no answer □ very unacceptable □ unacceptable □ neutral □ acceptable □ very acceptable

4. Mode of intake
How would you like to take your antipsychotic medication? *(multiple answers possible)*
□ daily tablets □ 1-2 tablets a week □ liquid/drops (daily) □ depot-injection (every 2-6 weeks)

5. Additional questions
Do you smoke? □ yes □ no
Did you ever have an epileptic insult? □ yes □ no
Are you pregnant, do you breast feed or do you want to in the future? □ yes □ no
